# Supplementary material for: Cerebellar Kv3.3 potassium channels activate TANK-binding kinase 1 to regulate trafficking of the cell survival protein Hax-1
Source: Nat Commun. 2021 Mar 19;12:1731. doi: 10.1038/s41467-021-22003-8 (PMC7979925; doi:10.1038/s41467-021-22003-8)
Supplement: Supplementary file 1 — Supplementary Information [file 41467_2021_22003_MOESM1_ESM.pdf]

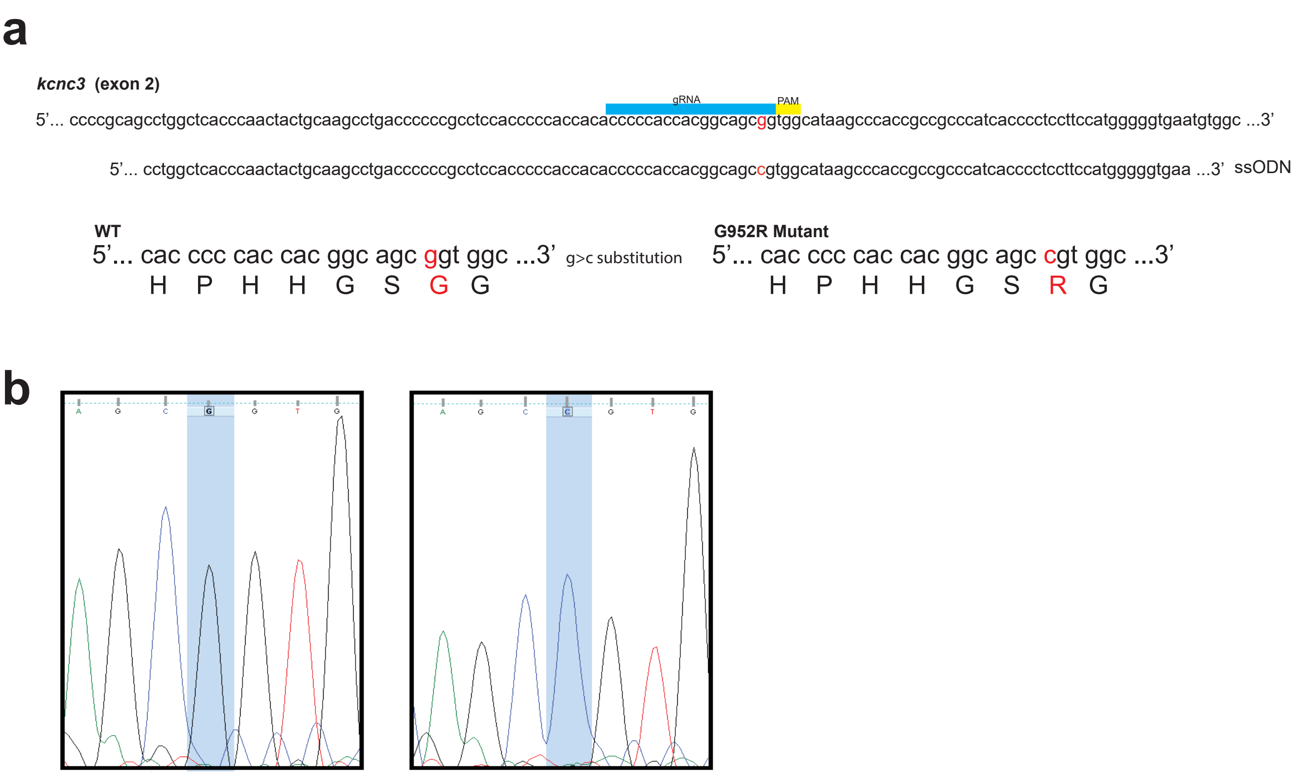

Supplementary Figure 1: Generation of G592R Kv3.3 knock-in mice. a, CRISPR/CAS9 targeting in mouse *Kcnc3* locus. The guide RNA and PAM sequences are highlighted in blue and yellow respectively, Amino acid sequences are shown under DNA sequences. The substituted nucleotides are shown in red. **b**, Sanger sequences indicating the mutation caused by the Cas9 mRNA and gRNA. Highlights show substituted amino acids.

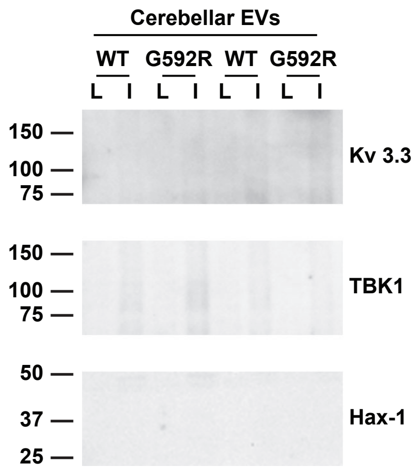

Supplementary Figure 2: Western blots for Kv3.3, TBK1 or Hax-1 in extracellular vesicles (EVs) isolated from cerebella of wild type and G592R Kv3.3 mutant mice. Low density fractions (L) and intermediate density fractions (I) were isolated from cerebella as described in legend to figure 8. Results representative of 3 independent experiments.
