# Supplementary material for: Cerebellar Kv3.3 potassium channels activate TANK-binding kinase 1 to regulate trafficking of the cell survival protein Hax-1
Source: Nat Commun. 2021 Mar 19;12:1731. doi: 10.1038/s41467-021-22003-8 (PMC7979925; doi:10.1038/s41467-021-22003-8)
Supplement: Supplementary file 3 — Reporting Summary [file 41467_2021_22003_MOESM3_ESM.pdf]

natureresearch

Corresponding author(s): Leonard K. Kaczmarek

Last updated by author(s): Jan 12, 2021

Reporting Summary

Nature Research wishes to improve the reproducibility of the work that we publish. This form provides structure for consistency and transparency in reporting. For further information on Nature Research policies, see Authors & Referees and the Editorial Policy Checklist .

Statistics

For all statistical analyses, confirm that the following items are present in the figure legend, table legend, main text, or Methods section.

n/a	Confirmed	
[ ]	[x]	The exact sample size (*n*) for each experimental group/condition, given as a discrete number and unit of measurement
[ ]	[x]	A statement on whether measurements were taken from distinct samples or whether the same sample was measured repeatedly
[ ]	[x]	The statistical test(s) used AND whether they are one- or two-sided<br>*Only common tests should be described solely by name; describe more complex techniques in the Methods section.*
[x]	[ ]	A description of all covariates tested
[ ]	[x]	A description of any assumptions or corrections, such as tests of normality and adjustment for multiple comparisons
[ ]	[x]	A full description of the statistical parameters including central tendency (e.g. means) or other basic estimates (e.g. regression coefficient) AND variation (e.g. standard deviation) or associated estimates of uncertainty (e.g. confidence intervals)
[ ]	[x]	For null hypothesis testing, the test statistic (e.g. *F*, *t*, *r*) with confidence intervals, effect sizes, degrees of freedom and *P* value noted<br>*Give P values as exact values whenever suitable.*
[x]	[ ]	For Bayesian analysis, information on the choice of priors and Markov chain Monte Carlo settings
[x]	[ ]	For hierarchical and complex designs, identification of the appropriate level for tests and full reporting of outcomes
[ ]	[x]	Estimates of effect sizes (e.g. Cohen's *d*, Pearson's *r*), indicating how they were calculated

Our web collection on statistics for biologists contains articles on many of the points above.

Software and code

Policy information about availability of computer code

Data collection	Whole cell voltage clamp recording data was acquired using Clamp 9 software (Molecular Devices, CA). Local field potentials were recorded using a 16-channel silicon probe (A1x16; NeuroNexus Technologies, Inc, Ann Arbor, MI). Electron Microscopy images were taken with Tecnai 12 Biotwin electron microscope. Keyence fluoresence microscope wwas used to collect fluoresence image data.
Data analysis	Whole cell volatge clamp data were analzed using offline Clampfit 10.6 software (pCLAMP 10.6; Molecular Devices, CA). Quanttative offline power spectrum analysis were performed using Matlab (Mathworks, Natick, MA). GraphPad Prism 7 and Image J 1.52i (Fiji) were used to analyze dat ain this study too.

For manuscripts utilizing custom algorithms or software that are central to the research but not yet described in published literature, software must be made available to editors/reviewers. We strongly encourage code deposition in a community repository (e.g. GitHub). See the Nature Research guidelines for submitting code & software for further information.

Data

Policy information about availability of data

All manuscripts must include a data availability statement. This statement should provide the following information, where applicable:

- Accession codes, unique identifiers, or web links for publicly available datasets
- A list of figures that have associated raw data
- A description of any restrictions on data availability

The data that support the findings of this study are available from the corresponding author upon reasonable request.

Field-specific reporting

Please select the one below that is the best fit for your research. If you are not sure, read the appropriate sections before making your selection.

[x] Life sciences [ ] Behavioural & social sciences [ ] Ecological, evolutionary & environmental sciences

For a reference copy of the document with all sections, see nature.com/documents/nr-reporting-summary-flat.pdf

Life sciences study design

All studies must disclose on these points even when the disclosure is negative.

Sample size	Sample size for each experiment was indicated in the figure legend. No statistical method was used to predetermine the sample size. We determine the sample size similar to previous studies (PMID: 26997484, PMID: 28119399, PMID: 23115170)
Data exclusions	No exclusion except that for mice that had health concerns.
Replication	Experiments were independently repeated, the numbers of replicates for each experiment were presented in the Figure legend.
Randomization	Age-matched mice were randomly assigned into control or experimental groups. Cell dishes were randomly assisnged to groups by investigators.
Blinding	Investigators were blinded where relevant. Technicians and collaborators do not know a particular outcome was expected.

Reporting for specific materials, systems and methods

We require information from authors about some types of materials, experimental systems and methods used in many studies. Here, indicate whether each material, system or method listed is relevant to your study. If you are not sure if a list item applies to your research, read the appropriate section before selecting a response.

Materials & experimental systems

n/a	Involved in the study	
[ ]	[x]	Antibodies
[ ]	[x]	Eukaryotic cell lines
[x]	[ ]	Palaeontology
[ ]	[x]	Animals and other organisms
[x]	[ ]	Human research participants
[x]	[ ]	Clinical data

Methods

n/a	Involved in the study	
[x]	[ ]	ChIP-seq
[x]	[ ]	Flow cytometry
[x]	[ ]	MRI-based neuroimaging

Antibodies

Antibodies used

Primary antibodies used in thsi study:
1. Polyclonal rabbit anti-Kv3.3 antibody (Alomone Labs, 1:200, #APC-102)
2. monoclonal rabbit anti-phospho TBK1 antibody (Cell Signaling Technology, 1:500, #5483)
3. monoclonal mouse anti-GAPDH antibody (Santa Cruz Biotechnology, 1:1000, #32233)
4. monoclonal rabbit anti-TBK1 antibody (Abcam, 1:1000, # ab40676)
5. Polyclonal anti-rabbit CD63 (Santa Cruz Biotechnology, 1:200, # sc-15363)
6. anti-pS6 antibody (Cell Signaling Technology, 1:1000, #2211)
7. cleaved caspase-7 (ASP198) (Cell Signaling technology, 1:500 , # 9491)
8. mouse monoclonal anti-LAMP2 antibody (Santa Cruz Biotechnology, 1:200, # sc-18822)
9. polyclonal anti-rabbit anti-LC3BII (Cell Signaling Technology, 1:500, # 2775S)
10. anti-Alix (Cell Signaling Technology, 1:1,000, #92880S)
11. anti-Annexin A2 (Abcam, 1:20,000, #ab178677)
12. anti-CD63 (Abcam, 1:1,000, #ab217345)
13. anti-Calbindin D28K (ThermoFisher Scientific, 1:1,000, #PA5-85669)
14. anti-Hax1 (Proteintech, 1:200, #11266-1-AP)
15. anti-HSC70 (Santa Cruz Biotechnology, 1:4,000, #sc-7298)
16. an Alexa 488 conjugated anti-NeuN antibody (Millipore Sigma, 1:1000, # MAB377X)
17. anti-Apo-B (1:1000, ab# 20737, abcam)
18. anti-Flotilin 1 (1:1000, #610821, BD Biosciences)
19. anti-GM130 (1:5000, # 610822, BD Biosciences)
20. anti-lamin-A/C (1:100, #sc-376248)

	21. anti-sec 61B (1:1000, #14648S, Cell Signaling Technology)<br><br>Secondary antibodies used in this study:<br>1. HRP conjugated anti-rabbit IgG, (ThermoFisher Scientific, 1:1000, # 32460),<br>2. HRP conjugated anti-mouse IgG (1:1000), (ThermoFisher Scientific, 1:1000, # 32430),<br>3. Alexa fluor 488 goat anti-rabbit secondary antibody (ThermoFisher Scientific, 1:1000, # A-11008),<br>4. Alexa fluor 594 goat anti-mouse secondary antibody (ThermoFisher Scientific, 1;1000, # A-11005).
Validation	All antibodies used are commerically available and were validated either by manufacture or used extensively in published research papers.

Eukaryotic cell lines

Policy information about cell lines

Cell line source(s)	CHO cells, Kv3.3 cells, Kv3.1 cells, BK cells
Authentication	The cell lines were generated in our lab and characterized previously (See PMID: 11160386, PMID: 26997484, PMID: 15375169).
Mycoplasma contamination	All lines were tested negative for mycoplasma.
Commonly misidentified lines (See ICLAC register)	No commonly misidentified cell lines were used.

Animals and other organisms

Policy information about studies involving animals; ARRIVE guidelines recommended for reporting animal research

Laboratory animals	wild type mice used in this study is B6. G592R Kv3.3 knock-in mice was generated using Crispr Cas9 gene editing technology (see method).
Wild animals	This study did not involve wild animals.
Field-collected samples	No Field-collected samples were used.
Ethics oversight	All experimenal procedures were approved by the Institutional Animal Care and Use Committee of Yale University.

Note that full information on the approval of the study protocol must also be provided in the manuscript.
